# Evaluating the Risk–Benefit Profile of Corticosteroid Therapy for COVID-19 Patients: A Scoping Review

**DOI:** 10.3390/pharmacy12040129

**Published:** 2024-08-22

**Authors:** Daniel Hsiang-Te Tsai, Emma Harmon, Jan Goelen, Heather E. Barry, Li-Yang Chen, Yingfen Hsia

**Affiliations:** 1 Centre for Neonatal and Paediatric Infection, St George’s University of London, London SW17 0RE, UK; htsai@sgul.ac.uk (D.H.-T.T.);; 2 School of Pharmacy, Institute of Clinical Pharmacy and Pharmaceutical Sciences, College of Medicine, National Cheng Kung University, Tainan 70101, Taiwan; 3 School of Pharmacy, Queens University Belfast, Belfast BT9 7BL, UK

**Keywords:** corticosteroids, COVID-19, SARS-CoV-2, randomised clinical trial

## Abstract

Background: The 2019 coronavirus (COVID-19) outbreak was declared a global pandemic in March 2020. It quickly spread across all continents, causing significant social, environmental, health, and economic impacts. During the pandemic, there has been consideration of repurposing and repositioning of medications, such as corticosteroids, for the treatment of hospitalised COVID-19 patients. Objective: To assess and summarise corticosteroid regimens used for hospitalised COVID-19 patients, focusing on dosage, route of administration, and clinical outcome from clinical trials. Methods: PubMed and Embase databases and the grey literature were searched to identify randomised controlled trials (RCTs) that evaluated the efficacy of corticosteroids in hospitalised patients with COVID-19 between January 2020 and January 2023. This scoping review was conducted in line with the PRISMA extension for scoping reviews (PRISMA-ScR) checklist. Key findings: A total of 24 RCTs were eligible for inclusion. There was variation in the steroid regimens used for treatment across COVID-19 trials. Despite the heterogeneity of included RCTs, the overall results have shown the benefits of improving lung function and a lower all-cause mortality rate in hospitalised COVID-19 patients treated with systematic corticosteroids. Conclusions: Corticosteroids have proven to be an effective treatment for COVID-19 patients in critical condition. However, comparative effectiveness studies should be conducted to assess the efficacy and safety of optimal corticosteroid treatment at the population level. Moreover, the global burden of long COVID is significant, affecting millions with persistent symptoms and long-term health complications. Thus, it is also necessary to evaluate the optimal steroid regimen for long COVID treatment.

## 1. Introduction

Coronavirus disease 2019 (COVID-19) is an infectious disease that initially emerged from China in late 2019, caused by Severe Acute Respiratory Syndrome Coronavirus 2 (SARS-CoV-2) [1,2]. The outbreak of the COVID-19 pandemic has had a devastating social, economic, and health impact, and it has infected millions of people worldwide [2]. Symptoms present as cough, fever, sore throat, headache, loss of taste and smell, and other influenza-like presentations [3]. People with COVID-19 may also be asymptomatic or develop only mild symptoms. However, a substantial number (37%) of patients experience severe disease that requires invasive mechanical ventilation [1].

The pathological progression in severe critical COVID-19 patients includes interleukin-mediated tissue responses, leading to cytokine dysregulation marked by elevated 3levels of C-reactive protein and Interleukin-6. Interleukin-6 induces shortness of breath, pulmonary fibrosis, and a reduction in oxygen saturation and results in the development of acute respiratory distress syndrome (ARDS) [4], multiple organ failure, and death [1,5,6]. In ARDS, inflammation damages alveolar–capillary membranes, resulting in increased lung permeability and the exudation of high-protein oedematous fluid into air sacs [7]. The SARS-CoV-2 viruses enter human cells by attaching to a specific protein, angiotensin-converting enzyme-2 receptor (ACE-2). This process causes inflammation in the lungs and makes blood vessels leakier, leading to fluid buildup in the lungs [8]. It has been estimated that 14–30% of hospitalised COVID-19 patients developed severe respiratory failure from ARDS, requiring intensive care and prolonged ventilatory support [1,9]. Respiratory failure is the leading cause of mortality in COVID-19 patients. Therefore, anti-inflammatory drugs (such as corticosteroids) might be beneficial in reducing the intensity of the inflammatory response to COVID-19 by preventing or mitigating the excessive release of cytokines and inflammatory mediators [6,10,11].

Corticosteroids have been widely used in diseases similar to COVID-19, including allergies, severe influenza, and community-acquired pneumonia, or previous coronavirus outbreaks, such as severe acute respiratory syndrome (SARS) or the Middle East Respiratory Syndrome (MERS) [1,12,13,14]. The Randomised Evaluation of COVID-19 Therapy (RECOVERY) trial has shown that dexamethasone reduced the mortality rate by one-third in patients who were receiving mechanical ventilation and by one-fifth in patients receiving oxygen without mechanical ventilation [1]. While several systematic reviews have been published to evaluate the efficacy of corticosteroids for COVID-19 treatment, emerging clinical trials within the substantial volume of COVID-19-related publications need to be considered [15,16,17]. The current guidelines on the corticosteroid regimen vary between countries. There remain uncertainties regarding the efficacy of corticosteroids in COVID-19 patients [1,18]. It is crucial to understand the optimal steroid regimen for COVID treatment, as it can significantly impact patients’ long-term outcomes in terms of particular dosages, formulations, and durations. The aim of this scoping review was to summarise the corticosteroid treatments in hospitalised COVID-19 patients from published clinical trials, aiming to assess the risk and benefits associated with their use.

## 2. Materials and Methods

We followed the PRISMA extension for scoping reviews (PRISMA-ScR) checklist using PubMed and Embase databases with the following search terms: (corticosteroid OR steroid) AND (hospitalized OR hospitalised OR hospitalization OR hospitalisation) AND (“coronavirus disease 2019” OR coronavirus OR COVID-19 OR SARS CoV-19). Details regarding the search strategies are presented in the Supplementary Materials (Files S1 and S2). Only studies published in the English language and within the period of January 2020 to January 2023 were included.

Studies published as an observational study, systematic review, meta-analysis, conference proceeding, and commentary were excluded. The grey literature was also searched to obtain the “WHO guideline on drugs for COVID-19” and National Institute of Health (NIH) of the United States of America (USA) guidelines. These guidelines are important and allow for harmonisation of treatment, ensuring the safe and appropriate use of medicines based on the principle of evidence-based healthcare. Two reviewers (E. Harmon and DHT. Tsai) independently reviewed the titles and abstracts of studies retrieved from the searches based on the inclusion and exclusion criteria.

The full-length articles were then retrieved for final evaluation and data extraction. Details of each eligible study were extracted, including numbers of participants, doses of corticosteroids, durations, and outcomes. No evaluation of the quality or risk of bias was carried out because scoping reviews are not intended to assess the quality of evidence. We performed a narrative synthesis to summarise patients’ characteristics, the steroid treatment (including dose, duration, and formulation), and clinical outcomes.

## 3. Results

**Figure 1:**
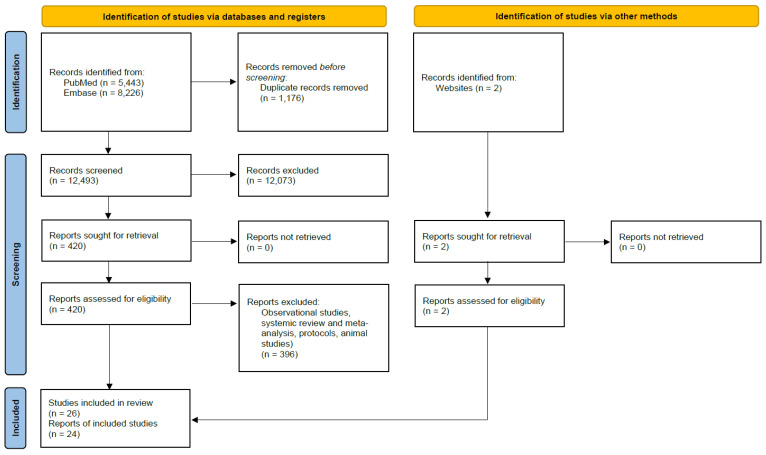
PRISMA 2020 flow diagram for new systematic reviews that included searches of databases and other sources.

This review included 24 papers after screening the titles and abstracts and removing duplicates. The results of the article screening and evaluation process are shown in the PRISMA flowchart in Figure 1. The PubMed database search identified 5443 papers and the Embase database identified 8226 papers. The published RCTs were conducted across Europe, the Americas, Southeast Asia, and the Eastern Mediterranean. Out of the 30 countries included, 8 were classified as high-income, and the remaining were classified as low- and middle-income based on the World Bank income classification.

Table 1 presents an overview of the study characteristics of the included studies. Eleven studies compared the efficacy of prednisolone, pulse methylprednisolone, hydrocortisone, and dexamethasone with standard care. Of these studies, eight studies focused on the efficacy of dexamethasone and pulse methylprednisolone in treating patients with COVID-19 with standard care, which refers to the current standard treatment for a particular disease or symptom in medical practice. One study investigated the efficacy of inhaled budesonide, and four studies examined the efficacy of high-dose dexamethasone (i.e., 8 mg thrice daily). The duration of steroid use varied between studies, ranging from 3 days to 28 days. This indicates the absence of a universal standard for healthcare professionals to follow. Ten studies reported the all-cause mortality rate for COVID-19 patients who received systematic corticosteroids for treatment. Several outcomes have been reported in these studies, including organ-support-free days, ventilator-free days, length of hospital stay, intensive care unit (ICU) admission, urgent care visits, recovery time, and clinical deterioration.

## 4. Discussion

Corticosteroids have been successfully used to treat patients with influenza-like diseases in the last few years. The duration of corticosteroids’ use affects their ability to reduce morbidity and mortality. The RECOVERY trial has shown a significant reduction in the 28-day mortality rate for COVID-19 patients whose symptoms had persisted for over 7 days compared to those with early-onset symptoms following dexamethasone therapy [1]. The current evidence primarily relies on RCTs, with limited research investigating steroid treatment at the population level. Uncertainty remains regarding the optimal timing for administering corticosteroid therapy due to insufficient clinical evidence [4]. This review summarised corticosteroid treatments for hospitalised COVID-19 patients from published clinical trials to provide a comprehensive assessment of the benefits and risk of their use.

RCTs are an essential study design in epidemiological research as they provide robust evidence of the efficacy and safety of medication use. Current studies and COVID-19 treatment guidelines do not recommend corticosteroid therapy for patients with mild symptoms of COVID-19 [1,13,36,37]. During the early stages of the COVID-19 pandemic, the WHO did not recommend the use of corticosteroids to treat COVID-19 patients who did not present with acute respiratory distress syndrome (ARDS) [25]. This was due to concerns of increasing viral replication and worsening disease progression, which may subsequently increase the mortality rate [6,36]. However, the RECOVERY trial has shown that dexamethasone treatment reduced the mortality rate for patients with COVID-19 on respiratory support [1]. The WHO and the National Institutes of Health (NIH) COVID-19 treatment guidelines were updated following the publication of supporting evidence from the RECOVERY trial. The use of corticosteroids has been recommended for hospitalised patients with COVID-19 requiring mechanical ventilation [38,39]. In addition, studies have shown that patients who received dexamethasone treatment along with standard care (the routine treatment typically given to patients) experienced a reduced time of invasive mechanical ventilation support compared to patients who only received standard care [2,21]. There was a higher all-cause mortality rate for COVID-19 patients who received higher doses and lower doses of dexamethasone. However, the dose regimen did not show any effect on the ventilator-free days.

Corticosteroid therapy has been shown to reduce ARDS-related inflammation, leading to improved respiratory rates and ventilation rates and a decreased need for mechanical ventilation and ICU treatment [4,40]. An Iranian study showed methylprednisolone pulse therapy (intravenous injection 250 mg/day for 3 days) was significantly effective and led to reduced mortality in hospitalised patients with severe COVID-19 compared to standard therapy (hydroxychloroquine sulphate, lopinavir, and naproxen, in accordance with the Iranian COVID-19 treatment protocol) [4]. Similarly, hydrocortisone treatment in severe COVID-19 patients showed an increase in organ-support-free days and reduced mortality within 21 days compared to outcomes in patients who only received standard care [19,26]. In addition, the treatment regimen including prednisolone for patients with moderate to severe COVID-19 symptoms was found to be superior in reducing the length of hospital stays compared to treatment without prednisolone [27]. Thus, there is compelling evidence that corticosteroid treatment plays a significant role in COVID-19 therapy based on RCTs.

Methylprednisolone has demonstrated greater efficacy in treating hospitalised COVID-19 patients compared to dexamethasone, as evidenced by improvements in clinical presentations, reductions in inflammatory markers, shorter recovery times and hospital stays, fewer ICU admissions, and lower mortality rates. The clinical benefit of using methylprednisolone to treat COVID-19 patients rather than dexamethasone has been shown in previous studies. One plausible explanation is that the increased immunosuppressive activity might be due to the deeper lung penetration of methylprednisolone and a greater affinity for the glucocorticoid receptor [7,33,34,41]. Also, the lower mortality rate with methylprednisolone 80 mg compared to dexamethasone 6 mg may be due to the higher equivalent dose (80 mg of methylprednisolone is roughly 15 mg of dexamethasone) [34,42].

Dexamethasone is more effective at reducing hospital stays and mortality for severe COVID-19 patients in intensive care compared to patients receiving inhaled budesonide for treatment. However, budesonide has demonstrated significant benefits in reducing urgent care visits in the early treatment of COVID-19 [32]. In addition, the combined use of different types of corticosteroids (e.g., adding dexamethasone therapy to methylprednisolone pulse therapy) should be reserved for extreme or life-threatening circumstances only [13]. For hospitalised patients with severe COVID-19, a short course of methylprednisolone for at least 5 days improved respiratory functional measures, including forced vital capacity. This suggests that long-term methylprednisolone treatment may be crucial for the long-term lung protection of hospitalised COVID-19 patients [20]. Other studies have shown that dexamethasone was more effective than methylprednisolone at improving clinical outcomes and shortening hospital stays [24,25]. While the evidence from RCTs supports the use of corticosteroids to treat hospitalised COVID-19 patients, uncertainties remain regarding which corticosteroid is most effective and the optimal dosage and duration.

Corticosteroids are associated with an increased risk of secondary infections, adverse effects, and other complications, which may outweigh their benefits [7,33]. The use of corticosteroids may increase the risk of developing systemic issues, such as autoimmune conditions, dyslipidaemia, or hypertension. In addition, there is a higher incidence of infections, delayed virus clearance, and avascular necrosis associated with corticosteroid therapy [23,42]. The use of corticosteroids, especially in diabetic and hypertensive patients, must be carefully weighed against the patient-specific risk–benefit ratio. Although diabetics on corticosteroids are at risk of hyperglycaemia due to impaired glycaemic control and insulin resistance [9], research has shown that corticosteroids can be safely used in COVID-19 management for diabetic patients at lower doses [27]. It has been reported that COVID-19 patients receiving mechanical ventilation and corticosteroid therapy have a high incidence of bacterial superinfections and pulmonary aspergillosis, but the underlying mechanism remains unclear [2].

Despite the prominent efficacy of dexamethasone in treating COVID-19, the optimal corticosteroid type, timing, dose, and duration have yet to be established [1]. The efficacy of corticosteroid therapy primarily depends on the severity of COVID-19 [27]. For patients with mild to moderate COVID-19 symptoms, high-dose administration of corticosteroids (i.e., 20 mg) did not show a reduction in mortality rate compared to those who received standard care alone [11]. A study has shown that patients with moderate to severe COVID-19 treated with higher doses of dexamethasone (8 mg three times daily) experienced more adverse events (e.g., hyperglycaemia, leucocytosis) and had a higher mortality rate compared to those who received a lower dose (8 mg once daily) [9]. Corticosteroid therapy is effective in hospitalised COVID-19 patients when administered at an appropriate dose and duration [27]. It is critical to consider the route of administration when giving corticosteroids to patients with COVID-19. Currently, no RCT has compared the efficacy of systemic corticosteroids administered through different routes (e.g., oral, parenteral, inhalation).

The strength of this review was to systematically review 24 studies, including types of corticosteroids, dosage regimens, mortality, and clinical outcomes. This review provides current insights into and practical recommendations for the use of corticosteroids for COVID-19 treatment in hospital settings. However, there are limitations to address. First, we restricted our searches to only papers published in the English language, which is a limitation of this review. We acknowledge that we did not include the use of corticosteroids in the treatment of patients with long COVID, referring to a range of symptoms that persist for weeks or months after the acute phase of a COVID-19 infection has resolved. It has been suggested that the inhaled long-acting bronchodilator (budesonide 400–800 µg/day) should be considered for patients with lung wheezing [43]. The global impact of long COVID is substantial, affecting millions with persistent symptoms and long-term health complications. Thus, it is necessary to evaluate the optimal steroid regimen for long COVID treatment.

Although COVID-19 vaccines have been used to successfully tackle the pandemic, severe acute respiratory syndrome coronavirus 2 (SARS-CoV-2) remains widespread globally. Future research efforts should explore the efficacy of corticosteroid use in treating COVID-19 among vulnerable populations, such as paediatrics, geriatrics, individuals with underlying health conditions, and pregnant or breastfeeding women [44]. It has been addressed that the efficacy of corticosteroid therapy for COVID-19 in these populations remains unclear [4,30]. In addition, a recent prospective observational cohort study in the UK examined the impact of corticosteroids on COVID-19 treatment. Patients aged 18 years or older with confirmed or highly likely COVID-19 who required supplementary oxygen were eligible for the study. The study revealed significant disparities in corticosteroid administration based on age, indicating that patients aged 70 years or older were less likely to receive corticosteroids, even considering illness severity, comorbidities, and clinical frailty [45]. Future research should explore barriers and potential health inequities related to corticosteroid use for COVID-19 treatment.

The COVID-19 pandemic began in early 2020, but evidence for corticosteroid use remains limited and primarily from RCTs. It is expected that more clinical trials will be conducted to investigate the efficacy of systematic corticosteroid use for patients with COVID-19 or long COVID symptoms. These studies will help determine the optimal use of corticosteroid therapy more effectively. There is a clear research gap, as the majority of RCTs recruited small numbers of patients and/or conducted no follow-up [4,30]. In addition, other factors, such as the administration route, the type of corticosteroids, the dosage, the duration, and the timing of administration, should be taken into consideration. Therefore, a larger sample size and a sufficient follow-up period are necessary to gain a better understanding of the efficacy of corticosteroid use in the treatment of COVID-19 patients, especially those who are severely and critically ill [20,24,35]. Furthermore, the appropriate doses of corticosteroids (e.g., dexamethasone and methylprednisolone) should be investigated for patients with COVID-19 and those experiencing long COVID symptoms [42].

## 5. Conclusions and Relevance

This review highlighted the clear benefits of systematic corticosteroid use as a safe and effective therapy for hospitalised patients with COVID-19. However, uncertainties remain regarding the optimal type of corticosteroid and the dosage regimen. The current evidence primarily relies on RCTs with small sample sizes. Future research should focus on evaluating the efficacy of systemic corticosteroid use for COVID-19 patients with underlying conditions. In addition, studies to assess the use of systemic corticosteroids for patients experiencing long COVID symptoms should be prioritised.

## Figures and Tables

**Table 1:** Study characteristics of included studies in the assessment of corticosteroid efficacy in hospitalised COVID-19 patients.

Author (Year)	Country	No. of Participants	Dose	Duration	Outcomes	Results
Angus (2020) [19]	Australia, Belgium, Canada, Croatia, Finland, France, Germany, Hungary, Ireland, Netherlands, New Zealand, Portugal, Romania, Saudi Arabia, Spain, United Kingdom, United States	576	(1) Fixed-dose HC: 50 mg, every 6 h (2) Shock-dependent HC: 50 mg, every 6 h while in shock	(1) Fixed-dose gr: 7 days (2) Shock-dependent gr: up to 28 days	Organ-support-free days within 21 days	(1) Fixed-dose gr OR: 1.43 (95% CrI 0.91–2.27) (2) Shock-dependent gr OR: 1.22 (95% CrI 0.76–1.94)
Barros (2021) [20]	Brazil	118	IV MP 0.5 mg/kg was given twice daily	5 days	Pulmonary function testing at day 120 follow-up visit	FEV_1_ and FVC were significantly higher in patients in the MP arm
Batirel (2021) [21]	Turkey	450	(1) High-dose: DEXA 6 mg/day equivalent (2) Pulse: 250 mg MP	10 days	ICU stay	(1) The pulse steroid gr had a shorter ICU stay (2) Median ICU stay: standard-care gr: 9.0 (95% CI 6.0–12.0) days; high-dose steroid gr: 8.0 (95% CI 5.0–13.0); pulse steroid gr: 4.5 (95% CI 3.0–8.0)
Bouadma (2022) [22]	France	546	(1) Standard: DEXA 6 mg/day (2) High-dose: DEXA 20 mg/day on days 1–5 and then 10 mg/day on days 6–10	10 days	60-day mortality	HR: 0.96 [95% CI, 0.69–1.33]
Corral-Gudino (2021) [23]	Spain	64	MP (40 mg twice daily for 3 days followed by 20 mg bid for 3 days)	6 days	A composite of death, admission to the intensive care unit, or requirement for non-invasive ventilation	(1) Standard of care gr: 14/29 (48%) (2) MP gr: 14/35 (40%)
Corral-Gudino (2023) [24]	Spain	125	(1) MP 250 mg/day (2) DEXA 6 mg/day	MP: 3 days DEXA: 10 days	28-day mortality	(1) Absolute risk difference, 0.1% [95% CI, −8.8 to 9.1%] (2) MP gr: 3 (4.8%) (3) DEXA gr: 3 (4.8%)
Dastenae (2022) [25]	Iran	143	(1) DEXA 8 mg/day (2) MP 60 mg/day in two divided doses	Maximum of 10 days	Length of hospital stay	(1) The duration of hospitalisation was significantly shorter in the DEXA gr (2) DEXA gr: 8 [95% CI: 6–10] (3) MP gr: 11 [95% CI: 7–14]
Dequin (2020) [26]	France	149	HC 200 mg/day until day 7 and then decreased to 100 mg/d for 4 days and 50 mg/day for 3 days	14 days	21-day mortality or respiratory support	(1) Hydrocortisone gr: 32/76 (42.1%) (2) Placebo gr: 37/73 (50.7%)
Edalatifard (2020) [4]	Iran	68	MP pulse 250 mg/day	3 days	Time of clinical improvement or death	The percentage of improved patients was higher in the MP group compared to the standard care group (94.1% versus 57.1%), and the mortality rate was significantly lower in the MP group (5.9% versus 42.9%; *p* < 0.001)
Ghanei (2021) [27]	Iran	336	Prednisolone 25 mg	5 days	ICU admission	No difference between treatment and placebo groups
Horby (2021) [1]	United Kingdom	6425	DEXA 6 mg once daily	Up to 10 days	28-day mortality	RR: 0.64 [95% CI, 0.51 to 0.81]
Jamaati (2021) [11]	Iran	50	DEXA 20 mg/day, days 1–5 and then 10 mg/day, days 6–10	10 days	Mortality	(1) Corticosteroid administration had no clinical benefit (2) DEXA gr: 64% (3) Placebo gr: 60%
Jeronimo (2020) [28]	Brazil	393	MP 0.5 mg/kg twice daily	5 days	28-day mortality	No difference between groups
Maskin (2022) [29]	Argentina	98	(1) DEXA 16 mg/day days 1–5 and then 8 mg days 6–10 or (2) DEXA 6 mg/day for 10 days	10 days	Ventilator-free days during the first 28 days	No difference between groups
Munch (2021) [30]	Denmark, Sweden, Switzerland, India	30	HC 200 mg/day	Up to 7 days	Number of days alive without life support on day 28	(1) No difference between groups (2) Hydrocortisone gr: 7 days (3) Placebo gr: 10 days
Pinzón (2021) [31]	Colombia	216	(1) DEXA 6 mg/day (2) MP 250 to 500 mg daily, days 1–3, and then prednisone 50 mg orally, days 4–17	(1) DEXA: up to 10 days (2) MP: 3 days and then prednisone 14 days	Recovery time	(1) Recovery time was shorter in the patients treated with MTP (2) MP gr: three days (3–4) (3) DEXA gr: six days (5–8)
Ramakrishnan (2021) [32]	UK	146	Budesonide turbuhaler 400 μg per actuation, two puffs to be taken twice per day	Median at 7 days (4–10)	COVID-19-related urgent care visits, including emergency department assessment or hospitalisation	(1) Difference in proportions 0·123, 95% CI 0·033 to 0·213; *p* = 0·009 (2) Budesonide gr: 2 (3%) (3) Olacebo: 11 (15%)
Ranjbar (2021) [33]	Iran	86	(1) MP 2 mg/kg/day (2) DEXA 6 mg/day	(1) MP: 10 days (2) DEXA: 10 days	28-day mortality rate	(1) No significant differences between groups (2) MP gr: 8 (18.6%) (3) DEXA gr: 15 (37.5%)
Salton (2022) [34]	Italy	677	(1) MP 80 mg daily infusion, days 1–8, and then slow tapering (2) DEXA 6 mg daily for up to 10 days	(1) MP: 8 days (2) DEXA: 10 days	28-day mortality rate	(1) No significant differences between groups (2) MP gr: 35 (10.4%) (3) DEXA: 41(12.1%)
Salvarani (2022) [13]	Italy	301	(1) MP: 1 g daily + standard treatment (2) Standard treatment: DEXA 6 mg/day oral or intravenous for 10 days	3 days	Duration of patient hospitalisation (median days)	(1) No significant differences between groups (HR, 0.92; 95%CI, 0.71–1.20) (2) MP gr: 15 (95% CI, 13.0 to 17.0) (3) Standard gr: 16 (95% CI, 13.8 to 18.2)
Soliman (2022) [7]	Egypt	67	(1) DEXA 8 mg/day (2) MP 1 mg/kg/day in two divided doses per day	7 days	Monitoring of systemic inflammation through follow-up of NLR ratio at days 5, 7	The NLR was significantly lower in the MP gr than the DEXA gr on the 5th and 7th days (*p*-values of 0.014 and 0.019, respectively)
Tang (2021) [35]	China	86	MP group 1 mg/kg per day	7 days	Incidence of clinical deterioration, 14 days	No significant differences between groups (4.8 vs. 4.8%, *p* = 1.000)
Tomazini (2020) [2]	Brazil	299	DEXA 20 mg once daily, days 1–5, and then 10 mg once, days 6–10 or until ICU discharge	10 days	Ventilator-free days during the first 28 days of hospital stay	(1) Difference, 2.26; 95% CI, 0.2–4.38; *p* = 0.04 (2) DEXA gr: 6.6 (95% CI, 5.0–8.2) (3) Placebo: 4.0 days (95% CI, 2.9–5.4)
Toroghi (2021) [9]	Iran	133	(1) Low-dose: DEXA 8 mg once daily (2) Intermediate-dose: DEXA 8 mg twice daily (3) High-dose: DEXA 8 mg thrice daily	Up to 10 days	60-day survival rate	Longer in the low-dose group than the high-dose group (HR = 0.36, 95% CI = 0.15–0.83)

Note: ICU: intensive care unit; IV: intravenous; OR: odds ratio; HR: hazard ratio; RR: risk ratio; gr: group; 95% CrI: 95% credible interval; 95% CI: 95% confidence interval; FEV_1_: forced expiratory volume in one second; FVC: forced vital capacity; HC: hydrocortisone; MP: methylprednisolone; DEXA: dexamethasone; NLR: neutrophil/lymphocyte.

## Data Availability

The original contributions presented in the study are included in the article/Supplementary Materials, further inquiries can be directed to the corresponding authors.

## Supplementary Materials

The following supporting information can be downloaded at: https://www.mdpi.com/article/10.3390/pharmacy12040129/s1, File S1: The search details in PubMed database; File S2: The search details in Embase database.
