# Supplementary material for: Evaluating the Risk–Benefit Profile of Corticosteroid Therapy for COVID-19 Patients: A Scoping Review
Source: Pharmacy (Basel). 2024 Aug 22;12(4):129. doi: 10.3390/pharmacy12040129 (PMC11360832; doi:10.3390/pharmacy12040129)
Supplement: Supplementary file 1 [file pharmacy-12-00129-s001.zip › pharmacy-3103694-supplementary.pdf]

Supplementary Materials

File S1. The search details in PubMed database

("adrenal cortex hormones"[MeSH Terms] OR ("adrenal"[All Fields] AND "cortex"[All Fields] AND "hormones"[All Fields]) OR "adrenal cortex hormones"[All Fields] OR "corticosteroid"[All Fields] OR "corticosteroids"[All Fields] OR "corticosteroidal"[All Fields] OR "corticosteroide"[All Fields] OR "corticosteroides"[All Fields] OR ("steroidal"[All Fields] OR "steroidals"[All Fields] OR "steroidic"[All Fields] OR "steroids"[MeSH Terms] OR "steroids"[All Fields] OR "steroid"[All Fields])) AND ("hospital s"[All Fields] OR "hospitalisation"[All Fields] OR "hospitalization"[MeSH Terms] OR "hospitalization"[All Fields] OR "hospitalised"[All Fields] OR "hospitalising"[All Fields] OR "hospitality"[All Fields] OR "hospitalisations"[All Fields] OR "hospitalizations"[All Fields] OR "hospitalize"[All Fields] OR "hospitalized"[All Fields] OR "hospitalizing"[All Fields] OR "hospitals"[MeSH Terms] OR "hospitals"[All Fields] OR "hospital"[All Fields] OR ("hospital s"[All Fields] OR "hospitalisation"[All Fields] OR "hospitalization"[MeSH Terms] OR "hospitalization"[All Fields] OR "hospitalised"[All Fields] OR "hospitalising"[All Fields] OR "hospitality"[All Fields] OR "hospitalisations"[All Fields] OR "hospitalizations"[All Fields] OR "hospitalize"[All Fields] OR "hospitalized"[All Fields] OR "hospitalizing"[All Fields] OR "hospitals"[MeSH Terms] OR "hospitals"[All Fields] OR "hospital"[All Fields]) OR ("hospital s"[All Fields] OR "hospitalisation"[All Fields] OR "hospitalization"[MeSH Terms] OR "hospitalization"[All Fields] OR "hospitalised"[All Fields] OR "hospitalising"[All Fields] OR "hospitality"[All Fields] OR "hospitalisations"[All Fields] OR "hospitalizations"[All Fields] OR "hospitalize"[All Fields] OR "hospitalized"[All Fields] OR "hospitalizing"[All Fields] OR "hospitals"[MeSH Terms] OR "hospitals"[All Fields] OR "hospital"[All Fields]) OR ("hospital s"[All Fields] OR "hospitalisation"[All Fields] OR "hospitalization"[MeSH Terms] OR "hospitalization"[All Fields] OR "hospitalised"[All Fields] OR "hospitalising"[All Fields] OR "hospitality"[All Fields] OR "hospitalisations"[All Fields] OR "hospitalizations"[All Fields] OR "hospitalize"[All Fields] OR "hospitalized"[All Fields] OR "hospitalizing"[All Fields] OR "hospitals"[MeSH Terms] OR "hospitals"[All Fields] OR "hospital"[All Fields])) AND ("coronavirus disease 2019"[All Fields] OR ("coronavirus"[MeSH Terms] OR "coronavirus"[All Fields] OR "coronaviruses"[All Fields]) OR ("covid 19"[All Fields] OR "covid 19"[MeSH Terms] OR "covid 19 vaccines"[All Fields] OR "covid 19 vaccines"[MeSH Terms] OR "covid 19 serotherapy"[All Fields] OR "covid 19 nucleic acid testing"[All Fields] OR "covid 19 nucleic acid testing"[MeSH Terms] OR "covid 19 serological testing"[All Fields] OR "covid 19 serological testing"[MeSH Terms] OR "covid 19 testing"[All Fields] OR "covid 19 testing"[MeSH Terms] OR "sars cov 2"[All Fields] OR "sars cov 2"[MeSH Terms] OR "severe acute respiratory syndrome coronavirus 2"[All Fields] OR "ncov"[All Fields] OR "2019 ncov"[All Fields] OR (("coronavirus"[MeSH Terms] OR "coronavirus"[All Fields] OR "cov"[All Fields]) AND 2019/11/01:3000/12/31[Date - Publication])) OR (("severe acute respiratory syndrome related coronavirus"[MeSH Terms] OR ("severe"[All Fields] AND "acute"[All Fields] AND "respiratory"[All Fields] AND "syndrome related"[All Fields] AND "coronavirus"[All Fields]) OR "severe acute respiratory syndrome related coronavirus"[All Fields] OR "sars"[All Fields]) AND "CoV-19"[All Fields]))

File S2. The search details in Embase database

('corticosteroid'/exp OR corticosteroid OR 'steroid'/exp OR steroid) AND (hospitalized OR hospitalised OR 'hospitalization'/exp OR hospitalization OR hospitalisation) AND ('coronavirus disease 2019'/exp OR 'coronavirus disease 2019' OR 'coronavirus'/exp OR coronavirus OR 'covid 19'/exp OR 'covid 19' OR 'sars cov-19' OR (('sars'/exp OR sars) AND 'cov 19'))
